# Effects of elevated CO_2_ on resistant and susceptible rice cultivar and its primary host, brown planthopper (BPH), *Nilaparvata lugens* (Stål)

**DOI:** 10.1038/s41598-021-87992-4

**Published:** 2021-04-26

**Authors:** Sengottayan Senthil-Nathan

**Affiliations:** Division of Biopesticides and Environmental Toxicology, Sri Paramakalyani Centre for Excellence in Environmental Sciences, Manonmaniam Sundaranar University, Alwarkurichi, Tenkasi, Tamil Nadu 627 412 India

**Keywords:** Chemical biology, Entomology

## Abstract

The elevated CO_2_ (*e*CO_2_) has positive response on plant growth and negative response on insect pests. As a contemplation, the feeding pattern of the brown plant hopper, *Nilaparvata lugens* Stål on susceptible and resistant rice cultivars and their growth rates exposed to *e*CO_2_ conditions were analyzed. The *e*CO_2_ treatment showed significant differences in percentage of emergence and rice biomass that were consistent across the rice cultivars, when compared to the ambient conditions. Similarly, increase in carbon and decrese in nitrogen ratio of leaves and alterations in defensive peroxidase enzyme levels were observed, but was non-linear among the cultivars tested. Lower survivorship and nutritional indices of *N. lugens* were observed in conditions of *e*CO_2_ levels over ambient conditions. Results were nonlinear in manner. We conclude that the plant carbon accumulation increased due to *e*CO_2_, causing physiological changes that decreased nitrogen content. Similarly, *e*CO_2_ increased insect feeding, and did alter other variables such as their biology or reproduction.

## Introduction

An increased amount of greenhouse gases due to human activities has been proposed to cause the global warming^1^. The magnitude of elevated CO_2_ (*e*CO_2_) levels has seriously impacted our environment by imposing a change on global climate^2–4^. Atmospheric CO_2_ is on an upsurge and has reached 409.46 ppm this year, 2018, from 407.18 in 2017^5^. The increase CO_2_ likely to alter the biology circuitously via climate change, and directly by creating changes in growth of plant growth, chemical composition of the plant tissue as well as influence on insect herbivory s life cycle^6^. Researchers have given much emphasis towards the effects of increased concentrations of CO_2_ are likely to have more impact on global climate. The increasing CO_2_ concentrations are also expected to have a direct effect on the growth, physiology, and chemistry of plants, independent of any effects on climate^7–9^. The utmost adverse effects of *e*CO_2_ on plants is an increase in the rate of photosynthesis, thereby increasing the carbon fixation by leaves. Higher photosynthetic rates eventually result in carbohydrate supply beyond demand for growth, that is stored in the plants, thus increasing the plant biomass, rather than their structural mass. In addition, the surplus carbon is engaged in the production of secondary metabolites^10^ and plant tissues like cell walls and organelles^11^. Besides these effects, reduction in transpiration rate and stomatal conductance^12^, suppression of dark respiration and photorespiration are also observed with higher CO_2_ levels^13^.

The quantity of food intake by insect on diverse host plants depends on the availability plant vigor which are known to be influenced by the carbon concentration in the surrounding environment that has direct effects on the plant quality factors such as plant biomass, water content and other plant traits such as leaf area, leaf thickness, chlorophyll content, carbon nitrogen (C and N) ratio in plant tissue and the secondary compounds^14,15^. The development, egg laying capacity, reproduction, adult longevity and population level of insect herbivores, may be affected due to the changes in host plant quality as well as quantity due to increased CO_2_ concentration which impacts plant performance. The C/N ratio suggests that the distribution of resources to secondary compounds is controlled by the carbon-nutrient status of a plant^11^.

The plants grown under increased CO_2_ levels are often characterized by lower nitrogen content, but with increased carbon-based secondary compounds^16^ without affecting the production of nitrogen-containing secondary compounds^11^ which is an expensive process requiring high enzymatic activity^11,17^. Supportive studies of the above statements include the reduced dietary quality of leaves due to reduced nitrogen (N) by 10–30% in plants grown in enriched CO_2_ conditions^18^. Lower foliar N content due to *e*CO_2_ has also been shown to cause an increase in food intake by the insects up to 40%^19^. Both the plant nutrient content and secondary metabolites influence insect herbivore performance^20^. Hence, insect growth and development has often been shown to be negatively correlated with *e*CO_2_^11,15^.

Many species of insects will meet less nutritious host plants under *e*CO_2_, which may bring both prolonged developmental times, greater larval mortality, and lower fecundity^21^. *E* levels of CO_2_ increase plant growth but may also increase the injury caused by some pest insects through increased feeding^22^.

Rice (*Oryza sativa* L. *Poaceae* Family) is the world's most significant crop for direct human consumption, feeding half of the world population. Recent studies indicated that *e*CO_2_ increased rice photosynthetic rates, growth rate, biomass, and grain yield^23^. The direct reaction of rice plant physiological increases is considered to be ‘positive’ responses to increased CO_2_ levels. The carbon-nutrient equilibrium recommends a premise that the carbon nutrient grade directly controls the secondary metabolite distribution in plants^11^.

The brown planthopper (BPH) *Nilaparvata lugens* (Stål) (Hemiptera: Delphacidae) and the white-backed planthopper, *Sogatella furcifera*, and are the important pests of rice in Asia^24–27^. Insect feeding patterns are varied among hopper species and showed either increased feeding rates or no feeding at all^14,15^. Herbivores may also influence plant productivity and the change metabolism rates by feeding on them. Increased feeding by herbivores in *e*CO_2_ regimes could potentially reduce plant productivity^28,29^. Under ambient ozone conditions, 5–35% reduction in crop yields was observed agriculturally important locations across South Asia which is about US$4 billion per annum for staple crops^30^.

Insects that fed on plants grown under *e*CO_2_ exhibited lower food utilization rates^21^. From this point of view, we investigated the response of susceptible and resistant rice varieties (IR 20 and ADT 46) under elevated and ambient CO_2_ on (1) emergence, root and shoot weight length, (2) estimation of defensive enzymes on different rice varieties, (3) biology and reproduction of *N. lugens* and (4) nutritional indices of *N. lugens* exposed to the plant varieties under elevate and ambient CO_2_ conditions.

## Results

### Effect of ambient and ***e***CO_2_ condition on emergence

**Figure 1:**
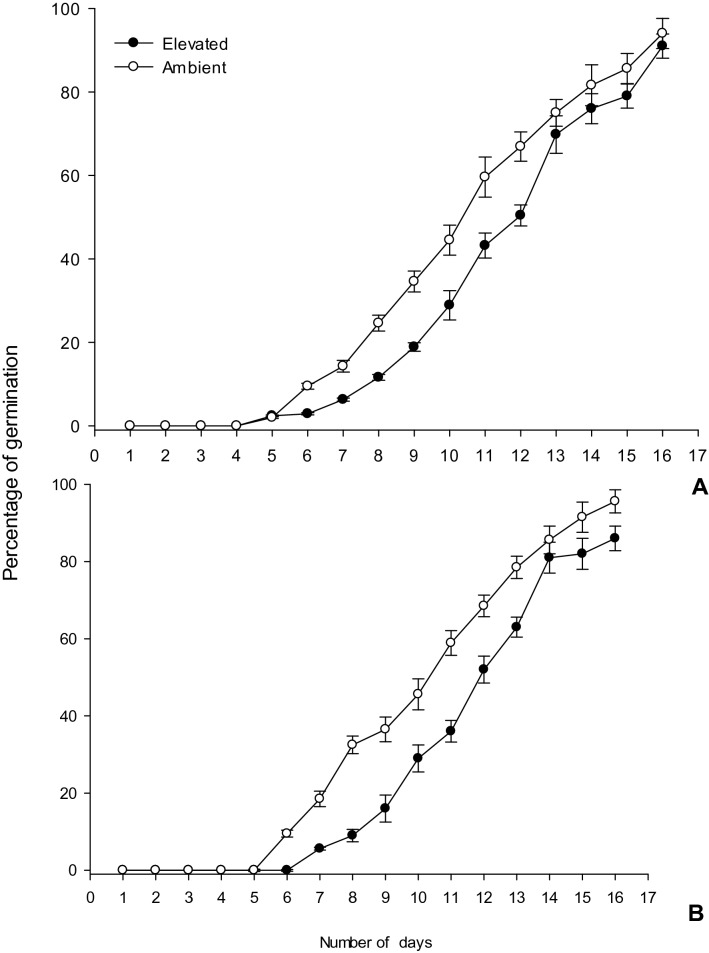
Percentage of different rice seed emergence under ambient and elevated condition. (**A**-IR 20; **B**-ADT 46).

The *e*CO_2_ increased early emergence rates on susceptible and resistant rice cultivar viz*.,* IR 20 and ADT 46, that was significantly different when compared plants grown under ambient CO_2_ conditions (χ^2^ = 9.7, d.f = 1, P = 0.003) consistently (Fig. 1A,B). A significant, 20% increase in the plant biomass (above and below ground) was observed as a result of plant exposure to *e*CO_2._ and was not influenced by the application of fertilizers.

**Figure 2:**
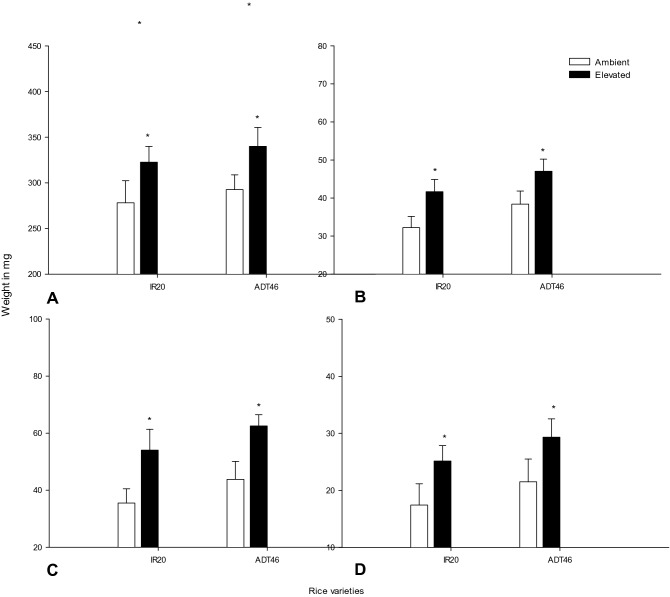
Effect of *e*CO_2_ on shoot (**A**–**B**) and root (**C**–**D**) weight of different rice varieties (Values are means ± SEM of five replications) (Mean (± SEM) followed by the symbol * in an individual experiment indicate significant difference (P < 0.05) in a Tukeys test) (ns-non significant).

Root-shoot ratio increased while grown in *e*CO_2_ condition rather than ambient condition. However, the increase in plant biomass and root-shoot ratio was inconsistent among the cultivars used in this study (Fig. 2). The effect of *e*CO_2_ on rice plants increased the shoot and root weight significant (*P* < 0.01) among the cultivars (Fig. 2A,B). For example, the IR 20 variety was influenced by *e*CO_2_ levels as it exhibited maximum shoot weight when compared with control (*F*_*1,8*_ = 15.89; *P* < 0.004). Most of the measured root and shoot length characteristics differed among rice varieties in both ambient and *e* condition. The *e*CO_2_ significantly increased shoot and tiller growth. The CO_2_ treatment across all rice varieties increased shoot growth for the rice.

The variances in the effect of the ambient and *e*CO_2_ effect on the root and shoot weight from glasshouse were analyzed (ANOVA) (Fig. 2) that showed a great deal of variation among ambience and elevation in speed and probability of seed germination. In general, *e*CO_2_ levels had a positive effect on the germination.

### Effect of ambient and *e*CO_2_ condition on carbon: nitrogen ratio

**Figure 3:**
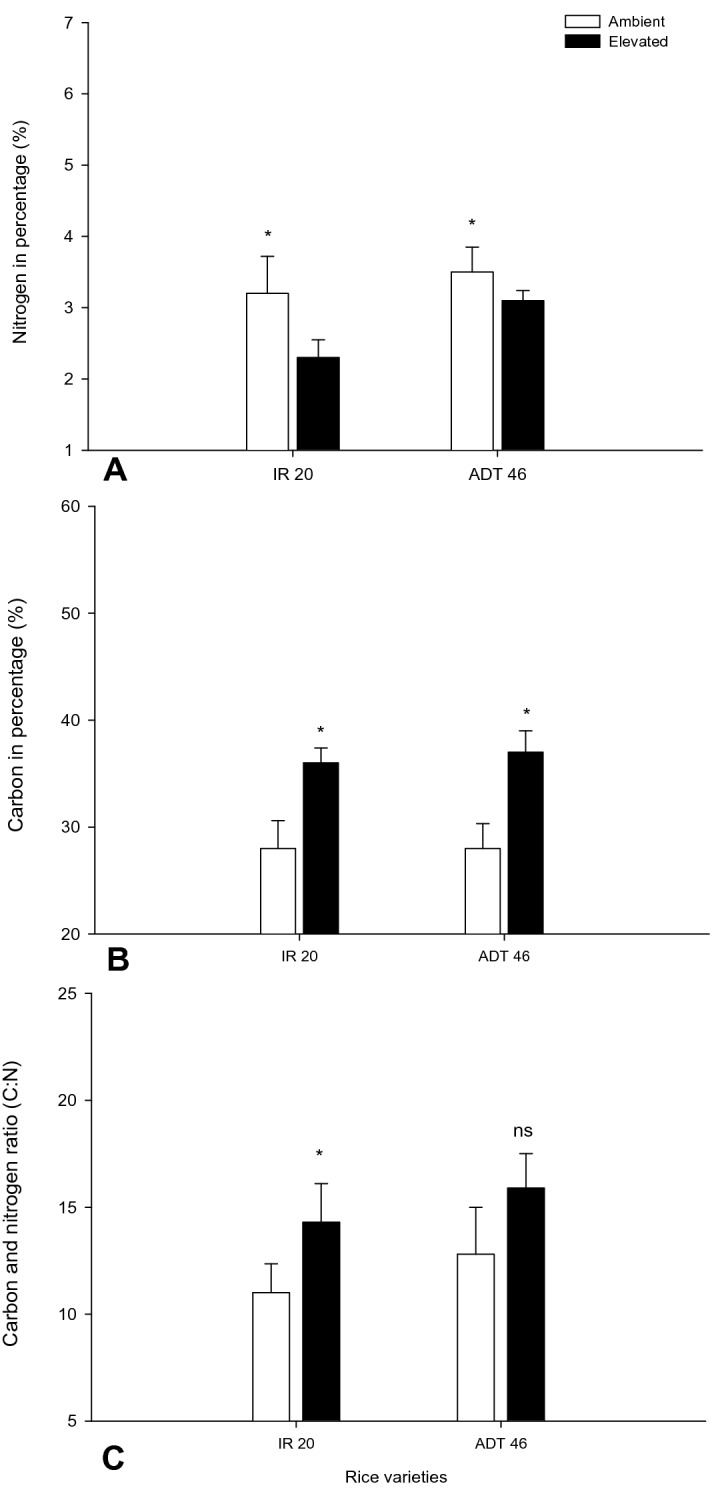
Effect of *e*CO_2_ on biochemical profile of different rice varieties (**A**- Nitrogen, **B**- Carbon, **C**- Nitrogen and Carbon ratio) (Values are means ± SEM of five replications) (Mean (± SEM) followed by the symbol * in an individual experiment indicate significant difference (P < 0.05) in a Tukeys test) (ns-non significant).

On average, *e*CO_2_ increased the C/N ratio of leaves by 5 to 7%, but the effect was not steady among the rice varieties (Fig. 3). Also, the effect was consistent among the cultivars (Fig. 3). But in the case of nitrogen, conflicting results were observed. Overall, nitrogen content in plants decreased in *e*CO_2_ condition compared to the ambient conditions. The nitrogen ratio shows greater change in rice strain ADT 46. We observed 1% of nitrogen decrease in rice strain ADT 46 when grown in *e*CO_2_ condition while it was significantly greater when compared with ambient grown ADT 46 (*F*_*1,8*_ = 7.10*; P* < 0.029). Nitrogen level in *e*CO_2_ condition was decreased in IR 20 rice leaves by 0.9% being significantly different (*F*_*1,8*_ = 7.76*; P* < 0.02) from ambient condition (Fig. 3A).

### Effect of ambient and *e*CO_2_ condition on rice plant growth

Under *e*CO2 condition (725 ppm), both of the rice varieties grew faster and attained maturity quicker, that was evident by the presence of senescent yellow leaf blades, compared with rice plants grown in ambient condition. The *e*CO_2_ influenced the rice plant physiology and growth, as yellow leaves per plant became greater than green leaves per plant, indicating changes in plant chemistry (data not shown).

### Effect of ambient and *e*CO_2_ condition on plant defense enzyme activity

**Figure 4:**
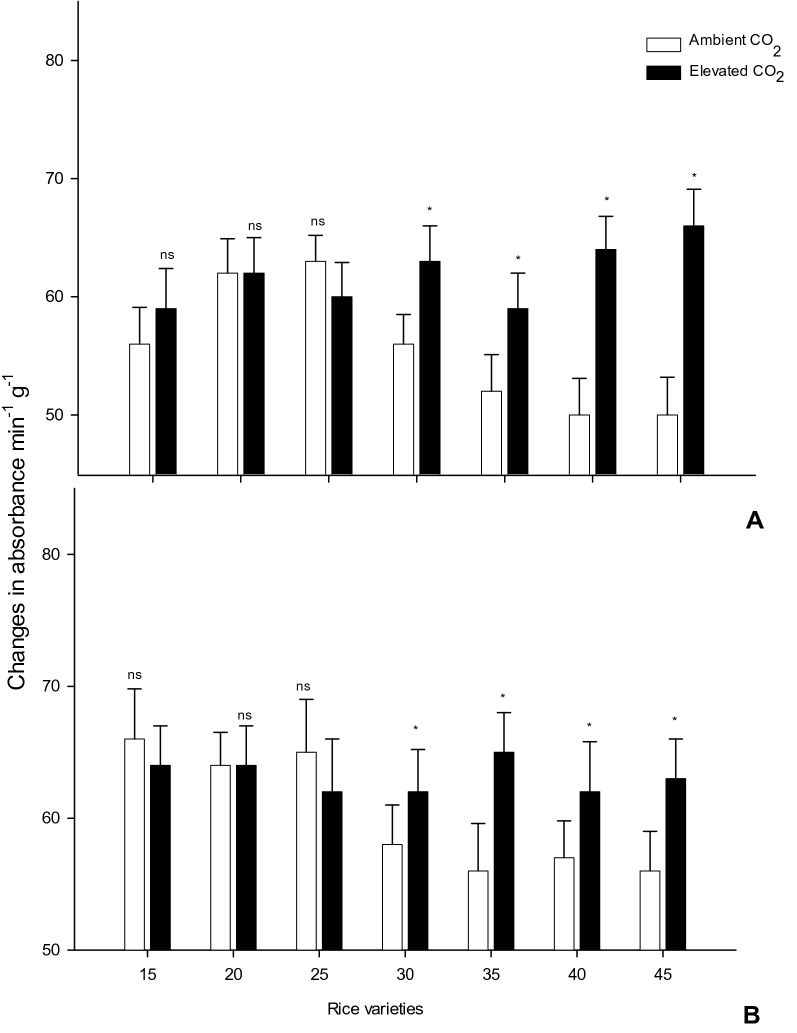
The peroxidase enzyme (PO) content in rice leaves grown in ambient and *e*CO_2_ (Values are means ± SEM of five replications) (Mean (± SEM) followed by the symbol * in an individual experiment indicate significant difference (P < 0.05) in a Tukeys test) (ns-non significant).

Peroxidase (POD) enzyme was activated more in *e*CO_2_ condition than ambient condition, 24 d postplanting (Fig. 4). Forty-five days post planting, the trend observed for *e*CO_2_ grown rice varieties changed to mimic the condition with the least enzyme activity as in ambient grown rice varieties. IR 20 rice variety grown in *e*CO_2_ had 6% more peroxidase activity than the same variety grown in ambient condition, after 45 days that was being significantly different (*F*_*1,8*_ = 0.79*; P* < 0.01). Similarly, ADT 46 rice varieties established increased peroxidase enzyme activity being 9% greater than rice grown in ambient conditions, after 24 d also being significantly different (*F*_*1,8*_ = 9.33*; P* < 0.012). However, at 45 days, ADT 46 rice variety was significantly different (*F*_*1,8*_ = 6.42*; P* < 0.005) from ambient grown rice plants at *e*CO_2_ which produced an increase of 11% enhanced peroxidase activity.

**Figure 5:**
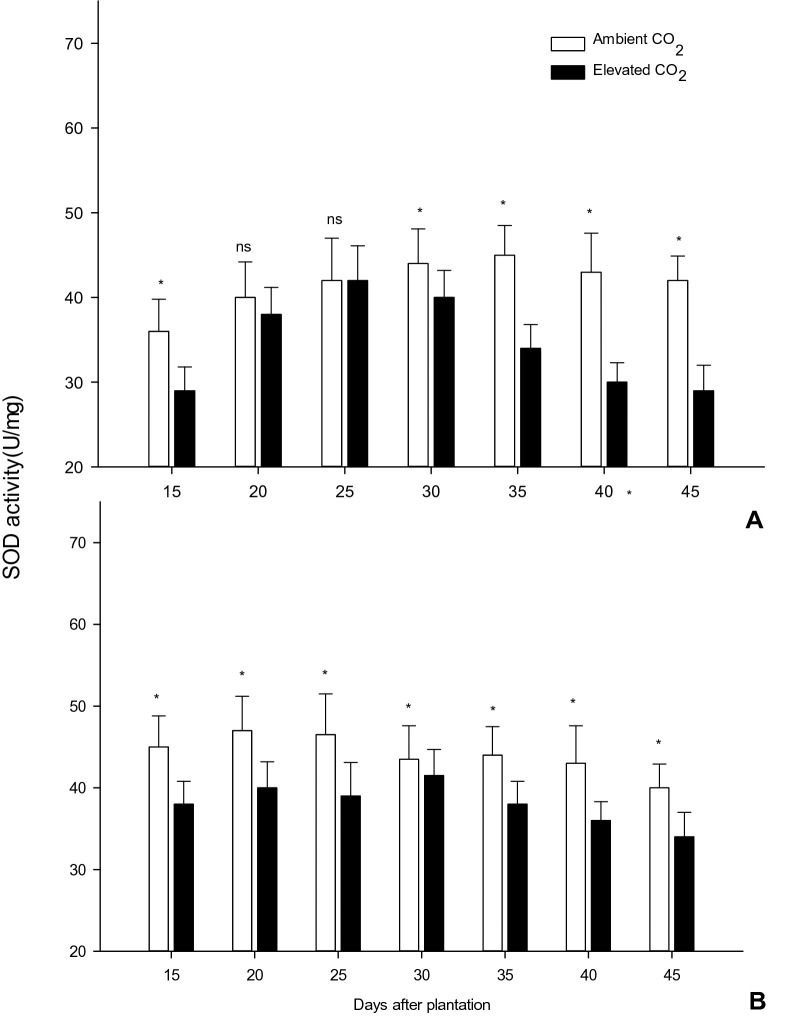
The Superoxide Dismutase (SOD) content in rice leaves grown in ambient and *e*CO_2_ (Values are means ± SEM of five replications) (Mean (± SEM) followed by the symbol * in an individual experiment indicate significant difference (P < 0.05) in a Tukeys test) (ns-non significant).

Superoxide dismutase (SOD) enzyme was decreased in activity of *e*CO_2_ condition than ambient condition, 24 d post-planting (Fig. 5). IR 20 rice variety grown in *e*CO_2_ had 10.8% less superoxide dismutase activity than the same variety grown in ambient condition, after 45 days that was being significantly different (*F*_*1,8*_ = 12.15*; P* < 0.008). Similarly, ADT 46 rice variety established decreased superoxide dismutase activity being 9.8% less than rice grown in ambient conditions, at 45 days, ADT 46 rice variety was significantly different (*F*_*1,8*_ = 13.94*; P* < 0.006) from ambient grown rice plants at *e*CO_2_ which produced a decrease of 11.8% of superoxide dismutase activity.

### Effect of ambient and *e*CO_2_ on *N. lugens* survival

**Figure 6:**
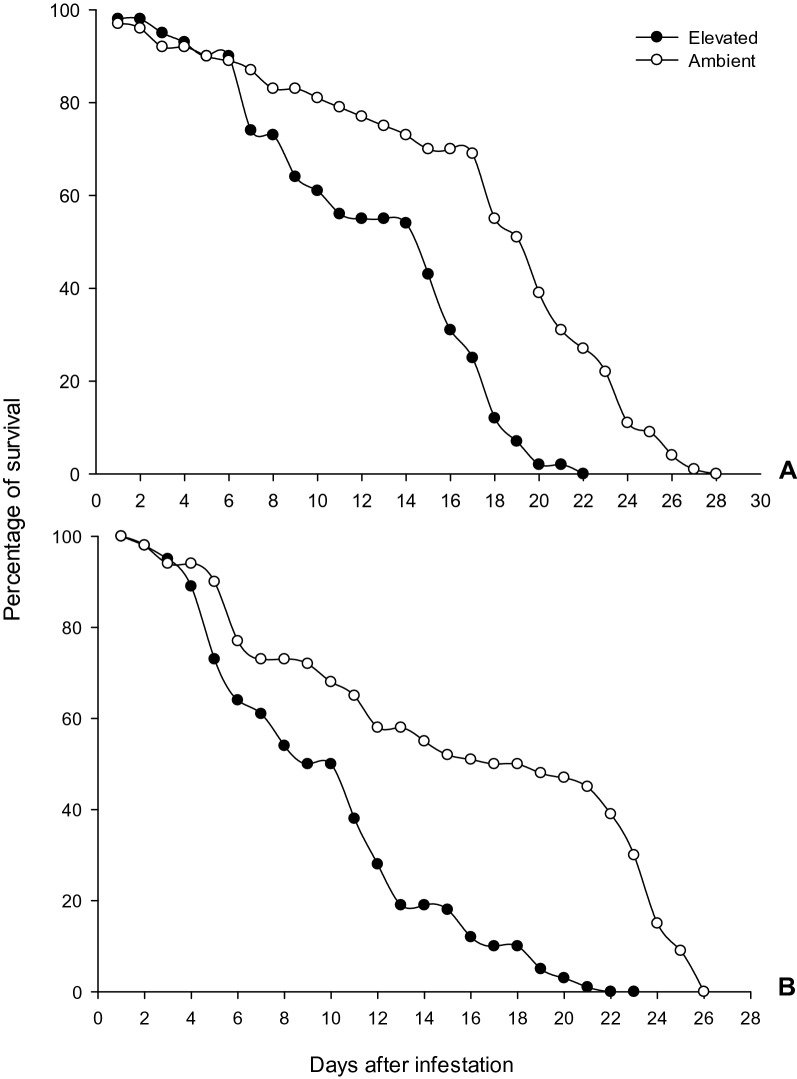
Survival rate of *N. lugens* on rice varieties (A-IR 20; B- ADT 46) grown in ambient and *e*CO_2_ condition. Survivorship curves differ at the α = 0.05 confidence interval according to log-rank statistics.

The *N. lugens* survivorship was significantly decreased under *e*CO_2_, compared with ambient CO_2_. *N. lugens* longevity was significantly reduced when fed on rice varieties IR 20 _(_*χ*^2^ = 3.81, df = 1, *P* = 0.039), and ADT 46 _(_*χ*^2^ = 4.32, df = 1, *P* = 0.054) (Fig. 6A,B). The survivability of *N. lugens* nymphs feeding on rice plants grown in ambient condition reached 50% in 13 days. In contrast, *N. lugens* nymphs feeding on rice plants grown in *e*CO_2_ decreased into less than 10% survivability in 20 days (Fig. 6).

### Effect of ambient and *e*CO_2_ on nutritional indices of *N. lugens*

**Figure 7:**
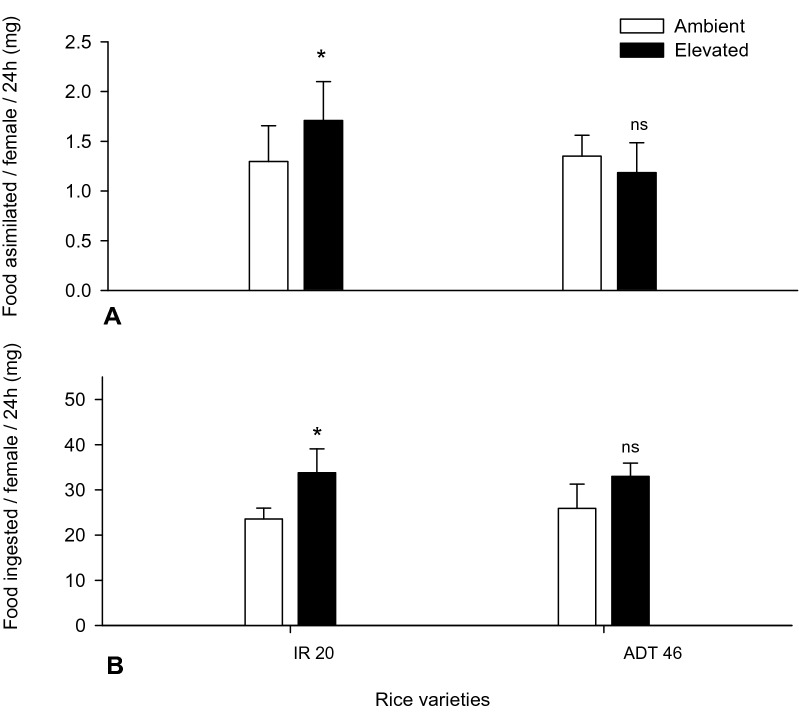
Food utilization of *N. lugens* under *e*CO_2_ (Mean (± SEM) followed by the symbol * in an individual experiment indicate significant difference (P < 0.05) in a Tukeys test) (ns-non significant).

The data presented in Fig. 7, revealed higher food assimilation and utilization rates of insects when grown in *e*CO_2_ conditions rather than ambient conditions at 24 h. Planthopper nymphs fed on plants grown in increased CO_2_ levels, exhibited increased food assimilation and ingestion. Efficiency of food conversion by females fed on rice leaves under *e*CO_2_ concentrations were reduced significantly (*F*_*5,10*_ = 17.94*, p* < 0.001) (by 25%) than when fed on ambient condition plants.

### Effect of ambient and *e*CO_2_ on life cycle of *N. lugens*

**Figure 8:**
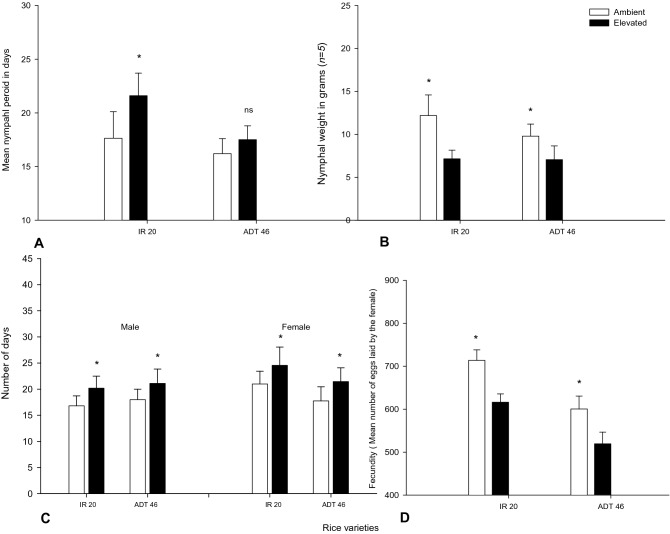
Mean nymphal period, nymphal weight and adult longevity of *N. lugens* in ambient and elevated condition (Mean (± SEM) followed by the symbol * in an individual experiment indicate significant difference (P < 0.05) in a Tukeys test) (ns-non significant).

Nymph development time was negatively influenced by the *e*CO_2_ and had negative influence on the development time of nymphs (*F*_*1,8*_ = 7.54*, p* < 0.025) in IR20 rice strain. However, it was not significantly different in the BPH nymph which fed on ADT46 rice strain (*F*_*1,8*_ = 3.83*, p* < 0.086) (Fig. 8A). Also, the *e*CO_2_ has negatively affecting their weight gain (Fig. 8B). The fresh weights of female adults were greatly decreased with the increase of CO_2_ content in the rice plants. The rice plants grown in *e*CO_2 strongly_ influenced fresh weights in the BPH both in IR20 (*F*_*1,8*_ = 79.73*, p* < 0.0001) and ADT46 (*F*_*1,8*_ = 17.56*, p* < 0.003). Females were significantly heavier when reared on the rice plants in ambient condition than in *e* condition (Fig. 8B).

Adult longevity of *N. lugens* increased when reared under *e*CO_2_ conditions (Fig. 8C,D). The longevity of adult male and females were significantly increased (*F*_*1,8*_ = 29.91*, p* < 0.001) with increase in CO_2_ content rice plants (Fig. 8C). Between generations, BPH reared on *e*CO_2_ times produced 10% less eggs (*F*_*1,8*_ = 19.70*, p* < 0.001) than those reared on ambient rice plants (Fig. 8D).

The results showed that *e*CO_2_ condition produced direct effects on the growth and development rates of *N. lugens* being decreased overall when feeding under *e* CO_2_ conditions.

## Discussion

Climate change resulting from increased carbon dioxide may play an important role in insect-plant interaction in several complex ways. Doubling of the current CO_2_ concentration modified the rate and extent of germination and emergence in rice species. When the seeds were produced under control conditions, a high CO_2_ concentration during germination of Arabidopsis thaliana seeds does not change the proportion of germinated seeds, but does induce faster germination. The response to [CO_2_] in germination seems to be species-specific, but on the whole positive, Wulff and Alexander^31^ found, in maternal lines of Plantago lanceolata, that both total germination and germination rate were stimulated by *e*CO_2_, whereas Garbutt et al*.*^32^ found no difference in germination rate in *Abutilon, Ambrosia*, *Chenopodium*, Amaranthus and *Setaria*^33^.

Given the overall high CO_2_ concentration in the soil, it is questionable whether a doubling in the atmospheric CO_2_ concentration would have any direct effect^34^. However, maternal effects have been reported, with a much lower germination rate for seeds from high CO^2^ parents of two Ipomoea species^35^ up to an increased germination rate in Pinus taeda^36,37^. Ziska and James^38^ has stated that one influence in the stimulation of germination and emergence may be ethylene. We did observe that the ethylene was not a toxin in the controlled-environment chambers. However, it is recognized that ethylene controls important components of germination, including enzyme secretion as well as cell expansion and differentiation. Some agronomists may vaccinate ethylene into the soil to stimulate weed germination^38^.

These results augment to the literature, which reports positive effects of *e*CO_2_ on the biomass of the rice. In this study the responses of the two rice varieties to *e*CO_2_ levels from did not differ substantially^39^. The effects of *e*CO_2_ and nutrient abundant on the aboveground biomass of the rice plants were additive^40^. This is in difference to the results of previous studies of Stöcklin and Körner^41^ and Matthies and Egli^42^, they proved stronger effects from *e*CO_2_ on plants growth and biomass especially root and shoot. Cohen et al*.*^43^ pointed out the N source and CO_2_ concentration affect the growth of root structure in terms of entire size and anatomy of diverse root orders. Root xylem growth was found to be sensitive to the N basis and also, although to a reduced extent, to the *e*CO_2._ Interactions involving carbon (C) and nitrogen (N) likely control terrestrial environment responses to *e*CO_2_ at scales from the leaf to the earth and from the second to the aera^44^. In particular, response to *e*CO_2_ may normally be lesser at low relative to high soil nitrogen supply and, in turn, *e*CO_2_ may impact on the soil nitrogen processes that control nitrogen availability to plants. Such responses could constrain the volume of terrestrial environments to obtain and store carbon under rising *e*CO2 levels^44^. Biochemical analysis of *Oryza sativa* revealed a substantial decrease (10%) of leaf carbon and nitrogen ratio under *e*CO_2_ conditions as opposed to ambient. The general increase of carbon and nitrogen ratio and the reduction in chlorophyll number of leaves under *e*CO_2_ supports the results published in earlier studies^15^. However, our study showed that effects differed among plant species. In addition, most of the insect pests seems to be undesirably affected by *e*CO_2_ because of the decrease in foliar nitrogen and increase in carbon and nitrogen ratio^15^. In our study, 15.9% increase in carbon and nitrogen ratio was observed under *e*CO_2_ conditions. It is well known that when plants are grown under *e*CO_2_ condition, they display increased rates of photosynthesis as well as lower N levels in leaves due to increased plant growth rates^45^.

This study reports on nutrient changes in carbon, nitrogen, peroxidase and super oxide dismutase of the rice varieties (IR 20 and ADT 46) when grown under increased CO_2_ conditions in rice plants. Peroxidase level was not significant up to 25 days while the rice plant were grown in amphient CO_2_ and *e*CO_2_. However, after 25 days to 45 it was significantly different. Tegelberg et al*.*^46^ observed that the activity of polyphenol oxidase (PPO) and guaiacol peroxidase (POD), from the leaves of six genotypes of silver birch *(Betula pendula* Roth) seedlings exposed to short-term *e*CO_2_ increased. Higher PPO activity suggests that in climate change conditions, phenolic substrates gradually leaked from apoplast to symplast. It is possible that the fast growth rate of the seedlings under *e*CO_2_ and amphient accelerated the onset of senescence in the fully expanded mature leaves activating also PPO^46,47^.

Along with plant physiology plant nutrition, disease tolerance and defense enzyme will also be affected/altered by temperature and CO_2_ level changes^48,49^. Superoxide dismutase (SOD) helps as a defensive enzyme formed in the cell to defend the damage of reactive oxygen in the biological evolution process; it could remove potentially unwanted superoxide anions and hydrogen peroxide, discharge the impairment to plant cells, and control lipid oxidation. A higher SOD value meant the plant experienced higher stress levels^50^. The results of the current study showed that the general SOD action decreased significantly in the *e*CO_2_ and was higher than that under ambient condition. This happened typically because the daily normal temperature increase in the heading stage was more than that of seedling and filling stages and the high temperature stress reduced the level of SOD activity^51^. Pritchard et al*.*^52^ was observed that *e*CO_2_ concentration (720 μL ^L−1^) on soybean (*Glycine max* (L.) Merr.) decreased the activities of superoxide dismutase (SOD, EC 1.15.1.1).

The majority of research, reports that an increasing temperature will result in a change in development times of many insects^53^. Temperatures also have a deliberate impact on the reproductive system of insects. The *e*CO_2_ levels influence the temperature, thereby exerting a direct influence on insect physiology. Furthermore, there was a strong correlation between *e*CO_2_, host plant with insect survival, weight gains, and development times^23^. Estimates predict that an increase in temperature from 1 to 3 °C leads to major changes in environmental conditions, which affect insect physiology. These changes can increase the number of insect generations per season. However, if environmental conditions extend or prolong developmental times, there may be fewer generations per season, while a shorter developmental time can increase the number of insect generations per season^48^. The survivorship of *N. lugens* nymphs decreased under *e*CO_2_ plants compared to control plants. The *e*CO_2_ condition of 750 ppm showed significant decrease in the survival rate. Xiao-Na et al*.*^54^ observed eCO_2_ (750 µl/l) on population abundances of *N. lugens* brachypterous-subpopulation significantly decreased (13.6%).

Native rice from most of the rice-growing areas of the world identified as highly resistant to the *N. lugens*. Wu et al*.*^55^ described that a high percentage of native rice strains were unaffected to the hopper population. The CO_2_ mediated changes in the rice foliage (i.e., decreased N and increased C) which affected the growth and development of *N. lugens*, causing higher consumption (55% in 350 ppm and 80% in 750 ppm CO_2_ condition). The increased larval weight (25 and 35%) with higher excreta material release was experienced under both *e*CO_2_ and ambient CO_2_. It is also observed that most phloem feeding insects display compensatory increase in food intake^56^. Insects, when fed on *e* and ambient CO_2_ grownup plants, were shown to increase their specific consumption due to the poor food quality of these plants^19,21^. In our study, the BPH development performance indices also significantly varied between *e*CO_2_ and ambient conditions. The relative growth rate of larvae fed (food assimilation and ingestion) on *e*CO_2_ plants was significantly reduced. Thus *N. lugens* consumed and assimilated more, but grew slower (lower relative growth rate), resulting in one to two days longer to reach pupation than when feeding on ambient plants. Food intake and digestion of the herbivore insects depend strongly on the nutritional quality of plant tissue^20^. However, our research was only aimed to explore the effects of changing food quality due to these factors on the feeding behavior and growth of insects as well as the defense strategies displayed by the host plants. *N. lugens*, when fed on resistant wild rice varieties were reported with various anomalies in settling, food consumption, absorption of ingested food, growth, lifespan, egg laying capacity and egg hatchability^50^. An increase in the rates of food ingestion and assimilation, irrespective of resistance and susceptible rice varieties were observed in N. *lugens* larvae fed with IR20. Other researchers made similar observations with *S. furcifera*, the white backed planthopper, which is a major, hemipteran pest of rice in Asia, evaluated on resistant cultivators^57^.

Adverse effects of resistant rice *O. punctata,* a diploid, which belongs to the *O. officinalis* complex within the Oryzeae genome groups, is a member of the BB genome type, which have also reported reduced hatchability of *N. lugens* eggs on resistant rice varieties^50^. Increased hopper egg laying and growth under *e*CO_2_ might also be allotted to advantageous microenvironment that results revealed from increased tillering and maximum plant growth under *e*CO_2_ conditions. In other hemipterans, *e*CO_2_ has been observed to increase egg lay like, cotton aphid, *Aphis gossypii*^58^; whitefly, *Bemisia tabaci*^59^, and peach aphid, *Myzus persicae* ^46,47,60,61^. Krishnan et al*.*^62^ projected that increasing CO_2_ levels would cause a reduction in yield, but an increase in CO_2_ level at each temperature increased yields, based on estimates from a two-crop simulation model.

## Conclusion

The effects of ambient (350 ppm) and *e*CO_2_ (725 ppm) levels on plant chemistry were evaluated. An increase in CO_2_ appears to be stimulate increases in plant growth under glasshouse conditions. Plant phytochemistry determined mainly by the independent effects of CO_2_ on rice plants causes slow growth and a decrease in yields. The results of this study suggest that an increase in CO_2_ causes an increase in rice growth, resulting in increases in biomass of the rice strains tested (IR 20 and ADT 46). However, the effect was not linear. Observations show that carbon content increased as nitrogen content decreased under increasing CO_2_. The total carbon nitrogen ratio decreased in ambient grown rice varieties. Defense related plant enzymes, peroxidase (PO) was increased and superoxide dismutase (SOD) activity was decreased significantly, 25 days post planting. The responses of Brown Planthopper (BPH) *Nilaparvata lugens* (Stål) feeding under *e*CO_2_ were variable and suggests that the effects on consumption and growth rate of this insect and other in the Hemiptera: Delphacidae, are not expected using the outline of the carbon-nutrient balance hypothesis. But the effects of CO_2_ and nutrient availability on insect pest dependent upon species. Survival rate of *N. lugens* decreased significantly in *e* CO_2_ as compared with ambient conditions. In *e*CO_2_ conditions the herbivores feeding level increases which correlates to an increase in plant growth. Finally this study suggests that CO_2_ increases the plant carbon accumulation while decreasing nitrogen content. Even though life span increase, but reproduction decrease in light of the trade-off mechanism.

## Methods

### Laboratory mass culture of *N. lugens*

The *N. lugens* culture is maintained in the laboratory of the SPKCES, M.S University, Alwarkurichi without any prior exposure to insecticide. These insects were maintained on *O. sativa* (IR 20) L. seedlings (nine to eleven days after germination (DAG) for first to third instar nymph; 21 DAG for late third instar nymph to adult) in acrylic cages.

### Glasshouse experiment

The glass house experiments were conducted for CO_2_ studies. The chambers (1 m × 0.5 m × 1.0 m) were sustained at 25 ± 2 °C and maximum 60% relative humidity. Daily daylight was supplemented by nine 400 W halide bulbs, positioned 0.3 m above the chambers (16-L: 8-D photoperiod). The level of CO_2_ inside the chamber controlled by using an infrared gas analyzer and 12 chambers (six-ambient CO_2_ at 350 ppm; six-*e* CO_2_ at 725 ppm) were set to circulate air from an external source. Concentration of CO_2_ was continuously monitored by the infra-red gas analyzer^63^.

### Experiment on the rice plant

Two rice varieties ‘IR 20- susceptible, conventional, and ADT 46- resistant conventional, semi-dwarf long grain used for this study. Both cultivars were generously provided by the Rice Research Station, Tamil Nadu Agricultural University, Ambasamudram, The cultivar IR 20 and ADT 46 used for research purpose only and both the rice cultivars does not come under endangered species of wild flora and fauna asper IUCN. Essential methods and guidelines are adopted from the IUCN.

The rice varieties were grown in a soil, six seedlings per pot were sown in and exposed to ambient and *e*CO_2,_ watered twice a week with tap water to ensure saturation. After germination, five pots per rice variety were used. In total, the experiment consisted of 30 pots per analysis and each pot contain two rice plants. Plants were randomly owed to glasshouse chambers of the same CO_2_ treatment after watering. Every week, the placement of the pots within the chambers were re- arranged to provide uniform experimental conditions.

### Effect of *e*CO_2_ on rice plant

For the biomass experiments, the rice plants were removed from the chambers after 5 weeks (36 days) and harvested for dry and wet weights measurement. The leaves were collected and counted randomly from each treatment. The collected plants were individually oven dried at 80 °C for 7 d, to measure their dry weight percentage. The nitrogen content was analyzed by using Kjehldahl procedure and carbon content was analyzed by using a CHN analyzer (Model Vario EL III).

### Percentage of emergence, root and shoot weight length and ratio estimation

The experiment was carried as described previously under ‘experiment on the rice plant’ in completely randomized design with alternate-day watering. Observations were recorded every day from the first day of germination after four days of sowing; ‘the first true-leaf arose after six days’. Seedlings were counted, cleaned, and upper-part and root fresh weight (mg/plant) as well as the root length (cm/plant) and the greater leaf length (cm) were determined. Percentage of emergence (PE) was calculated according to the formula 1^64^.

1 $${\text{MGT}} = \frac{{{\text{Dn}}}}{{\text{N}}}n$$

*‘*where: *‘n’* is the number of seeds that had germinated on day *D* and *D* is the number of days counted from the beginning of germination’.

### Estimation of defense enzymes

Ambient and *e*CO_2_ grown rice plants under glasshouse condition were examined. Five replications were sustained in each treatment; each replicate contain of five pots and in each pot four plants were maintained. For biochemical assays the leaf tissue was taken from the fifteenth day (15, 20, 25, 30, 35, 40 and 45d) after grown in ambient and *e*CO_2_ condition.

### Estimation of peroxidase (PO) activity

Hammerschmidt et al*.*^65^. protocol was followed for estimating the PO activity. Konn weight (1 g) of the rice leaves was homogenized in 2 ml of 0.1 M sodium phosphate buffer (pH 6.5) and centrifuged at 10,000×*g* for 25 min at 4 °C. The upperpart of the supernatant was used as an enzyme source. The enzyme extract (100 µl) was taken along with 1.5 ml of pyrogallol (0.05 M). To initiate the response, 100 ml of hydrogen peroxide (1%) (v/v) was supplemented to the sample cuvette and the absorbance was read at 420 nm (Lambda 25, UV/Vis spectrometer, PerkinElmer). The enzyme activity was expressed as change in absorbance min^-1^ g^-1^ fresh tissue.

### Estimation of superoxide dismutase (SOD) activity

Giannopolitis and Reis^66^ procedure was following to determine the SOD activity. Known quantity (1 g) of the plant leaves was homogenized in 2 ml of 0.1 M sodium phosphate buffer (pH 6.5) and centrifuged at 10,000 × g for 25 min at 4 °C. The enzyme extract (100 µl) was taken with nitro blue tetrazolium (NBT) in a reaction medium containing 50 mM potassium phosphate (pH 7.8), 14 mM methionine, EDTA 0.1 µM, NBT 75 µM and riboflavin 2 µM. The samples were illuminated for 7 min under 20 W. The spectrophotometer analysis was done in 560 nm, where one unit of SOD was measured as the amount of enzyme able to inhibit by 50% the photoreduction of NBT under the experimental conditions. The SOD activity was expressed in U mg^-1^ of protein.

### Biology and reproduction of *N. lugens*

Immature and mature insect of the *N. lugens* were fed on rice plants grown with ambient and *e*CO_2_. Daily mortality and number of eggs laid by insects were noted every day^67,68^.

To estimate the effects of CO_2_ treatment on percentage of egg hatched, 5 pairs of newly hatched brachypterous males and females were caged on 20-day-old caged plants. Each treatment was replicated for five times. The total number of nymphs that emerged denoted the number of viable eggs produced by the females. At the end of nymphal emergence, unhatched eggs were recorded by separating leaf sheaths under stereo microscope ^69^. Average lifetime number of eggs laid by the female and average daily eggs laid by the female was analyzed by analysis of variance (ANOVA) using Minitap 16 statistical software package (Minitap, State College, PA).

### Food utilization of BPH

To determine the intake of ingested and assimilated food, newly hatched BPH females that had been starved for three hours were evaluated individually on a microbalance. Each BPH was placed within a sealed parafilm sachet on the stem of twenty-five-day-old test plants. After 24 h, the BPH female and excreta weighed. The following formula (2) was used to estimate the food utlilization^57^.

2 $${\text{Food}}\,{\text{assimilated}} = {\text{IW}} \times \frac{{{\text{IC}} - {\text{FC}}}}{{{\text{IC}}}} + {\text{FW}} - {\text{IW}}$$

“where: IW = initial weight of test insect, FW-finial weight of test insect, IC-initial weight of control insect FC = final weight of control insect; and food ingested = food assimilated + weight of excreta”. There were four replications for each treatment including the control and the experiments were repeated three times for accuracy.

### Population growth index

The population growth index was estimated by following method. Twenty-five-day-old caged rice plants of control and CO_2_ treated were infested with five pairs of BPH per experimental cage. Each treatment was replicated five times. Nymphs and adults were counted 30 days after infestation^70,71^.

Mature and immature insects life span were analyzed using a log-rank χ2 test of equality over strata (PROC LIFE Table) along with formula (3) with Minitap16 statistical software package (Minitab 17, State College, PA).

3 $${\text{Nymph}}/{\text{Adult}}\,{\text{growth}}\,{\text{index}} = \frac{{{\text{Percent}}\,{\text{survival}}\,{\text{of}}\,{\text{nymph}}/{\text{adult}}}}{{{\text{Duration}}\,{\text{of}}\,{\text{nymph}}/{\text{adult}}}}$$

### Statistical analysis

Biology and nutritional indices were recorded as the mean of four replications and normalized by arcsine-square root transformation of percentages. The transformed percentages were undergone to analysis of variance (ANOVA). Differences between the five treatments were determined by Tukey’s–Kramer HSD test (P = 0.05) by using Minitab 17 software package. Population growth index data’s were analyzed using a log-rank χ2 test (PROC LIFE Table) with Minitab 17 statistical software package (Minitab, State College, PA).

## Data availability

The datasets generated during and/or analyzed during the current study are not publicly available due to funding agency agreement and intellectual properties but are available from the corresponding author on reasonable request with permission of funding agency.
